# Ongoing measles outbreak in Romania: Clinical investigation and molecular epidemiology performed on whole genome sequences

**DOI:** 10.1371/journal.pone.0317045

**Published:** 2025-01-15

**Authors:** Robert Hohan, Marius Surleac, Victor Daniel Miron, Andreea Tudor, Ana-Maria Tudor, Oana Săndulescu, Ovidiu Vlaicu, Victoria Aramă, Daniela Pițigoi, Adriana Hristea, Anca Cristina Drăgănescu, Dimitrios Paraskevis, Leontina Bănică, Dan Oțelea, Simona Paraschiv

**Affiliations:** 1 National Institute for Infectious Diseases “Prof. Dr. Matei Bals”, Bucharest, Romania; 2 Research Institute of the University of Bucharest, University of Bucharest, Bucharest, Romania; 3 Carol Davila University of Medicine and Pharmacy, Bucharest, Romania; 4 Department of Hygiene, Epidemiology and Medical Statistics, School of Medicine, National and Kapodistrian University of Athens, Athens, Greece; Universitas Syiah Kuala, INDONESIA

## Abstract

**Aim:**

Romania is currently facing a prolonged measles outbreak. The aim of the study was to analyse the circulating human measles virus (HMV) strains by combining whole genome sequencing (WGS) with phylogenetic analysis, with a focus on the haemagglutinin gene.

**Methods:**

We conducted an observational study in the first five months of 2024, in which 168 patients diagnosed with measles were randomly included. We have evaluated the clinical and epidemiological differences between children and adults. Screening for samples to be sequenced was performed with a commercial kit (PrimerDesign). WGS was done on Illumina MiSeq platform and phylogenetic analysis was performed with ML FastTree.

**Results:**

No significant epidemiological and clinical differences between patients in the two age groups were identified. WGS was successfully performed for a number of 124 HMV strains. Genotype analysis indicated all the sequences as D8, except one that was B3. Phylogenetic analysis identified two well supported clusters, suggestive for at least two local transmission networks in Romania. One large transmission network (n = 108) consisted of sequences both from adults and children. Only one sequence from outside Romania (reported in Russia in 2023) clustered within this group. Another small transmission cluster was identified (14 sequences of which 11 from patients infected in the spring of 2024 and three in 2022). A few differences between the two co-circulating viral variants/clusters were observed. The median duration of hospitalisation was 2 days longer for patients in smaller cluster compared to those in the larger one (p = 0.019). Furthermore, these two clusters presented different mutation profiles in the hemagglutinin (HA) and neuraminidase (N) genes with implications for molecular surveillance.

**Conclusion:**

The current measles epidemic in Romania is driven mainly by two D8 genotype variants with different mutation profiles and slightly different severity of the disease, highlighting the usefulness of sustained molecular surveillance.

## Introduction

After the COVID-19 pandemic, the public health in several countries is facing a new human measles outbreak, the main countries affected (by incidence per million inhabitants) being Azerbaijan, Kyrgyzstan, Kazakhstan, Armenia and Romania [1]. A significant increase in the number of cases has also been reported in Italy [2], France and Austria [3]. The high incidence rate is correlating well with a low vaccination rate in the countries characterised by a coverage below the recommended World Health Organization (WHO) 95% target for measles elimination [4]. Near half of the new cases in Romania were reported in susceptible children under 5 years of age correlated with the low uptake rate of the measles, mumps and rubella (MMR) vaccine in the paediatric population [5]. Romania has recently faced another measles epidemic (2016–2019) with significant numbers of cases and deaths; the majority in MMR unvaccinated children [6]. Since 2010, a continuous declining trend of MMR vaccination was observed in Romania and achieved the lowest threshold in 2022, with just 71.4% of children receiving 2 doses [7]. COVID-19 pandemic influenced vaccination uptake in different ways: increased vaccine hesitancy affected also MMR, pandemic restrictions impacted visits to general practitioners and lead to missed vaccination doses [7].This current outbreak started in 2023, with 21,133 cases and 21 deaths having been reported by mid July 2024 [8].

Human Measles Virus (HMV) is a single-stranded RNA virus that infects the target cells by interaction of hemagglutinin (HA) with CD150/CD46 receptors followed by membrane fusion [9,10]. Comparable to other RNA viruses, HMV evolves mainly through mutations induced by RNA dependent RNA polymerase, an enzyme that is error prone. Another important factor that shapes this virus evolution is the selective pressure exerted by the immune system. Neutralizing antibodies primarily target HA, accounting for about 90% of the antibodies [11]. Both vaccination and natural infection provide long-term immunity [12]. Of the 24 known HMV genotypes, D8 and B3 have dominated European outbreaks in the last decade [13,14]. Initial molecular analyses focused on a small genomic region, limiting insights into transmission chains and viral evolution [15]. Phylogenetic analysis is an important tool that proved its contribution in understanding the epidemic dynamics, identifying migration patterns, reconstructing the evolutionary history of viral pathogens [16–19]. This study aims to characterize the circulating HMV strains by the means of molecular epidemiological analysis performed on whole genome sequencing (WGS) and to assess the viral evolution by looking in particular at the HA gene structure. Different phylogenetic analyses on sub-genomic regions were performed in order to determine the phylogenetic signal and the genetic informative potential of the main viral genes. Clinical and epidemiological differences between children and adults were also evaluated.

## Methods

### Study population and definition of terms

We conducted an observational study among hospitalised patients diagnosed with measles at the National Institute of Infectious Diseases “Prof. Dr. Matei Bals” Bucharest. We included the patients hospitalised between January and May 2024 with a positive measles IgM that had a nasopharyngeal swab and blood sample collected in the first 24 hours after admission. A questionnaire containing demographic and epidemiologic data, chronic conditions, and clinical characteristics was completed. Throughout hospitalization, all signs and symptoms were closely monitored and documented. Any signs or symptoms that emerged during the hospital stay were recorded as present. Laboratory tests were performed on samples collected at the time of admission. Any deviation of parameter values from the normal laboratory range was classified accordingly as an increase or decrease. Measles IgM testing was performed with SERION ELISA classic Masern/Measles virus IgM (Serion Diagnostics, Wurzburg, Germany).

All patients received an ophthalmic examination by a specialist physician, who made the diagnosis of conjunctivitis and/or keratitis. The diagnosis of measles pneumonia was based on clinical evaluation, which included symptoms such as cough and dyspnea and suggestive findings on chest X-ray. Chest X-ray was performed only on the recommendation of the physician and was not a mandatory study procedure. Patients with respiratory symptoms requiring supplemental oxygen were classified as having acute respiratory failure. The diagnosis of acute hepatitis was established according to previous definitions [20] for patients with aminotransferase levels exceeding five times the laboratory’s normal value. Depending on the clinical course, other complications were identified and classified through specific investigations and assessments. Based on age, patients were categorised into two groups: children, if they were under 18 years at the time of admission, and adults. Retrospectively, three available nasopharyngeal specimens from patients with a diagnosis of measles in 2022 were included and the medical records were reviewed similarly to the prospectively included patients.

This study adheres to the workplace guidelines regarding conducting scientific research and publishing the results and was approved by the Ethical Committee of the National Institute for Infectious Diseases ‘Matei Bals’ (approval number C09810/2024). All patients in the study provided written informed consent.

### Real Time PCR testing and WGS

Nasopharyngeal swabs collected on viral transport media (VTM) were used to extract nucleic acids with High performance total nucleic acids extraction kit (MagNA Pure, Roche) and MagNA Pure instrument (Roche). Real-time RT-PCR was performed by using Human Measles Virus Genesig Advanced Kit (PrimerDesign). Samples with Ct values below 30 were further processed for WGS as described previously [21]. The DNA library was sequenced on MiSeq platform (Illumina).

### WGS reads assembly and phylogenetic analysis

The raw reads were mapped on reference human measles virus genome (AB016162.1) by using Geneious. Consensus sequences covering full genome or near full genome were successfully generated for 124 samples. In order to identify phylogenetic relatedness between Romanian HMV sequences from this outbreak and other sequences reported in other geographical area, we have performed Blast analysis [22]. Different analyses were performed on partial genomic regions: nucleocapsid gene, fusion and haemagglutinin genes. Phylogenetic analysis was performed with FastTree as implemented in the Geneious software using the Generalized Time Reversible model of nucleotide evolution, with a Gamma20-based likelihood. Refined analysis was performed on WGS set using BEAST software package [23]. The trees were visualised using FigTree v.1.4.4 [24].

### Mutations, selective pressure, structural implications

Mutations in HA were evaluated by comparing the Romanian sequences with vaccine and subtype D8 reference sequences. Synonymous and non-synonymous mutations were identified. Variation at the level of nucleic acid and protein was assessed by using Shannon Entropy-Two tool [25]. The 3D Protein Data Base (PDB) structures of *Measles morbillivirus* hemagglutinin glycoprotein have been downloaded from RCSB database based on structural domains sequence-related information from UniProt database [26]. The PDB structures used were: 2RKC, 2ZB5, 2ZB6, 3ALW, 3ALX, 3ALZ and 3INB. The PDB structures contain monomers (2RKC, 2ZB5, 2ZB6, 3ALW, 3ALZ), dimers (3INB) or tetramers (3ALX) [27]. The structures have been analyzed in PyMol software.

The sequences were aligned with primers and probe sequences recommended by the United States Centers for Disease Control and Prevention for measles detection, targeting N gene (CDC, Atlanta) [28].

### Statistical analysis

All analyses were conducted using SPSS (version 25.0, IBM, USA) [29]. Categorical variables were reported as frequencies and percentages, with comparative analysis performed using chi-square tests and odds ratio (OR), accompanied by 95% confidence intervals (95% CI). For continuous variables, we reported either the median with interquartile range (IQR, 25th and 75th percentiles) or the mean with standard deviation. Comparisons of continuous variables were made using the Mann-Whitney U test. Statistical significance was determined by p<0.05.

### Nucleotide sequence accession numbers

The WGS raw data generated in this study can be accessed under BioProject PRJNA1145141 (https://www.ncbi.nlm.nih.gov/bioproject/1145141, https://www.ncbi.nlm.nih.gov/sra/?term=PRJNA1145141).

## Results

### Epidemiological and clinical analyses of the studied patients

A total of 168 patients, with a median age of 8 years (IQR: 2, 33.8 years; range: 1 month to 56 years), were included in the analysis. Paediatric patients constituted 62.5% (n = 105), including 75 children under 5 years. In order to observe differences between children and adults, we have tried to include in this study as many adults as possible, reaching ratio children vs. adults of 1.7:1. According to the national data, the calculated case ratio of children vs. adults was 6:1 [8]. The patients were from 11 counties in southern Romania (Bucharest, Ilfov, Argeș, Târgoviște, Giurgiu, Dâmbovița, Prahova, Buzău, Ialomița, Călărași, Constanța). The epidemiological link was identified in 29.2% of cases. The majority were either unvaccinated (70.2%) or had unknown (do not remember) vaccination status (19.0%). Detailed statistical analysis comparing children and adults is provided in Table 1.

**Table 1:** Epidemiological, clinical and outcome characteristics of study patients.

Characteristics	All patients, n (%)	Children, n (%)	Adults, n (%)	p-value	OR	95%CI
	N = 168	N = 105	N = 63			
**Epidemiological data**						
Female	87 (51.8)	52 (49.5)	35 (55.6)	0.449	0.78	0.41–1.46
Urban	109 (64.9)	60 (57.1)	49 (77.8)	**0.007**	0.38	0.18–0.77
Epidemiological link	49 (29.2)	32 (30.5)	17 (27.0)	0.630	1.18	0.59–2.37
**Measles vaccination status**						
Unvaccinated	118 (70.2)	94 (89.5)	24 (38.1)	**<0.001**	NA	NA
Incompletely vaccinated (1 dose)	10 (6.0)	6 (5.7)	4 (6.3)			
Fully vaccinated (2 doses)	8 (4.8)	3 (2.9)	5 (7.9)			
Unknown	32 (19.0)	2 (1.9)	30 (47.6)			
**Chronic conditions**						
At least one chronic disease	19 (11.3)	2 (1.9)	17 (27.0)	**<0.001**	0.05	0.01–0.20
Cardiovascular disease	10 (6.0)	0 (0.0)	10 (15.9)	NA	NA	NA
Lung disease	4 (2.4)	0 (0.0)	4 (6.3)	NA	NA	NA
Neurological condition	4 (2.4)	1 (1.0)	3 (4.8)	0.117	NA	NA
Liver disease	4 (2.4)	0 (0.0)	4 (6.3)	NA	NA	NA
Obesity	4 (2.4)	1 (1.0)	3 (4.8)	0.117	NA	NA
Cancer	2 (1.2)	0 (0.0)	2 (3.2)	NA	NA	NA
HIV infection	2 (1.2)	0 (0.0)	2 (3.2)	NA	NA	NA
Pregnant women^a^	2 (1.2)	0 (0.0)	2 (3.2)	NA	NA	NA
**Clinical features**						
Fever	167 (99.4)	105 (100)	62 (98.4)	0.195	NA	NA
Cough	161 (95.8)	103 (98.1)	58 (92.1)	0.058	4.43	0.83–23.61
Runny nose	123 (73.2)	101 (96.2)	22 (34.9)	**<0.001**	47.1	15.3–145.0
Rash	168 (100)	105 (100)	63 (100)	1.000	NA	NA
Koplick spots	133 (79.2)	90 (85.7)	43 (68.3)	**0.007**	2.79	1.30–5.97
Red eyes	141 (83.9)	99 (94.3)	42 (66.7)	**<0.001**	8.25	3.10–21.90
Sore throat	56 (51.4)^b^	16 (34.8)^b^	40 (63.5)	**0.003**	0.31	0.14–0.68
Headache	31 (28.4)^b^	4 (8.7)^b^	27 (42.9)	**<0.001**	0.13	0.04–0.40
Dyspnea	27 (16.1)	15 (14.3)	12 (19.0)	0.416	0.70	0.31–1.62
Myalgia	23 (21.1)^b^	3 (6.5^b^	20 (31.7)	**0.001**	0.15	0.04–0.54
Diarrhoea	68 (40.5)	56 (53.3)	12 (19.0)	**<0.001**	4.85	2.32–10.14
Nausea and vomiting	30 (17.9)	21 (20.0)	9 (14.3)	0.349	1.50	0.63–3.51
**Laboratory findings**						
Increased WBC	6 (3.6)	5 (4.8)	1 (1.6)	0.283	3.10	0.35–27.16
Decreased WBC	39 (23.2)	24 (22.9)	15 (23.8)	0.887	0.94	0.45–1.98
Increased neutrophils	4 (2.4)	1 (1.0)	3 (4.8)	0.117	0.19	0.02–1.89
Decreased neutrophils	18 (10.7)	15 (14.3)	3 (4.8)	0.053	3.33	0.93–12.01
Decreased lymphocytes	111 (66.1)	49 (46.7)	62 (98.4)	**<0.001**	0.01	0.002–0.11
Decreased platelets	38 (22.6)	10 (9.5)	28 (44.4)	**<0.001**	0.13	0.06–0.29
Increased C-reactive protein	36/38 (94.7)	31/31 (100)	5/7 (71.4)	**0.002**	NA	NA
Increased ALT	91 (54.2)	33 (31.4)	58 (92.1)	**<0.001**	0.04	0.01–0.10
Maximum ALT increase^c^, median (IQR)	4.0 (2.1, 7.6)	1.8 (1.2, 4.9)	4.9 (3.0, 9.9)	**<0.001**	NA	NA
Increased AST	132 (78.6)	73 (69.5)	59 (93.7)	**<0.001**	0.15	0.05–0.46
Maximum AST increase^c^, median (IQR)	1.9 (1.3, 4.5)	1.3 (1.1, 1.8)	3.8 (2.2, 6.2)	**<0.001**	NA	NA
Increased CPK	41 (44.6)	13 (31.7)	28 (54.9)	**0.026**	0.18	0.08–0.37
Maximum CPK increase^c^, median (IQR)	2.5 (1.7, 5.1)	2.2 (1.5, 3.2)	2.8 (1.8, 7.6)	0.193	NA	NA
**Complications**						
At least one complication	155 (92.3)	100 (95.2)	55 (87.3)	0.062	2.91	0.91–9.32
Conjunctivitis	131 (78.0)	94 (89.5)	37 (58.7)	**<0.001**	6.00	2.70–13.37
Keratitis	35 (20.8)	20 (19.0)	15 (23.8)	0.462	0.75	0.35–1.61
Measles acute pneumonia	61 (36.3)	53 (50.5)	8 (12.7)	**<0.001**	7.00	3.04–16.14
Acute respiratory failure	20 (11.9)	10 (9.5)	10 (15.9)	0.219	0.56	0.22–1.42
Acute hepatitis	36 (21.4)	8 (7.6)	28 (44.4)	**<0.001**	0.10	0.04–0.25
Myositis	41 (44.6)	13 (31.7)	28 (54.9)	**0.026**	0.17	0.08–0.37
Acute dehydration	75 (44.6)	71 (67.6)	4 (6.3)	**<0.001**	30.8	10.33–91.8
Pericarditis	2 (1.2)	2 (1.9)	0 (0.0)	NA	NA	NA
Laryngitis	7 (4.2)	7 (6.7)	0 (0.0)	NA	NA	NA
Pancreatitis	1 (0.6)	0 (0.0)	1 (1.6)	NA	NA	NA
Acute otitis acute	2 (1.2)	2 (1.9)	0 (0.0)	NA	NA	NA
Acute sinusitis	1 (0.6)	0 (0.0)	1 (1.6)	NA	NA	NA
Days of hospitalization, median (IQR)	5 (4, 6)	5 (4, 6.5)	4 (4, 6)	0.080	NA	NA

NA–not applicable; IQR–interquartile range (25th, 75th percentile); ALT–alanine aminotransferase; AST–aspartate aminotransferase; CPK—Creatine phosphokinase.

^a^—Pregnancy was not considered a chronic condition

^b^—These symptoms were collected only in patients over 2 years of age. The denominator was modified, N = 109 for all patients and N = 46 for children

^c^—The increase was calculated in relation to the maximum value of the lab normal range by age and sex for each patient.

Complications were present in 92.3% of patients, with conjunctivitis being the most common, especially in children. Children had a higher risk of acute dehydration (30.8-fold) and measles pneumonia (6.9-fold), while adults had a 9.7-fold higher risk of acute hepatitis. Acute respiratory failure occurred in 11.9% of cases, equally distributed between children and adults. The median hospitalization duration was 5 days (IQR: 4–6), with no significant age group differences. No deaths were reported.

### Phylogenetic analysis of HMV sequences

**Fig 1:**
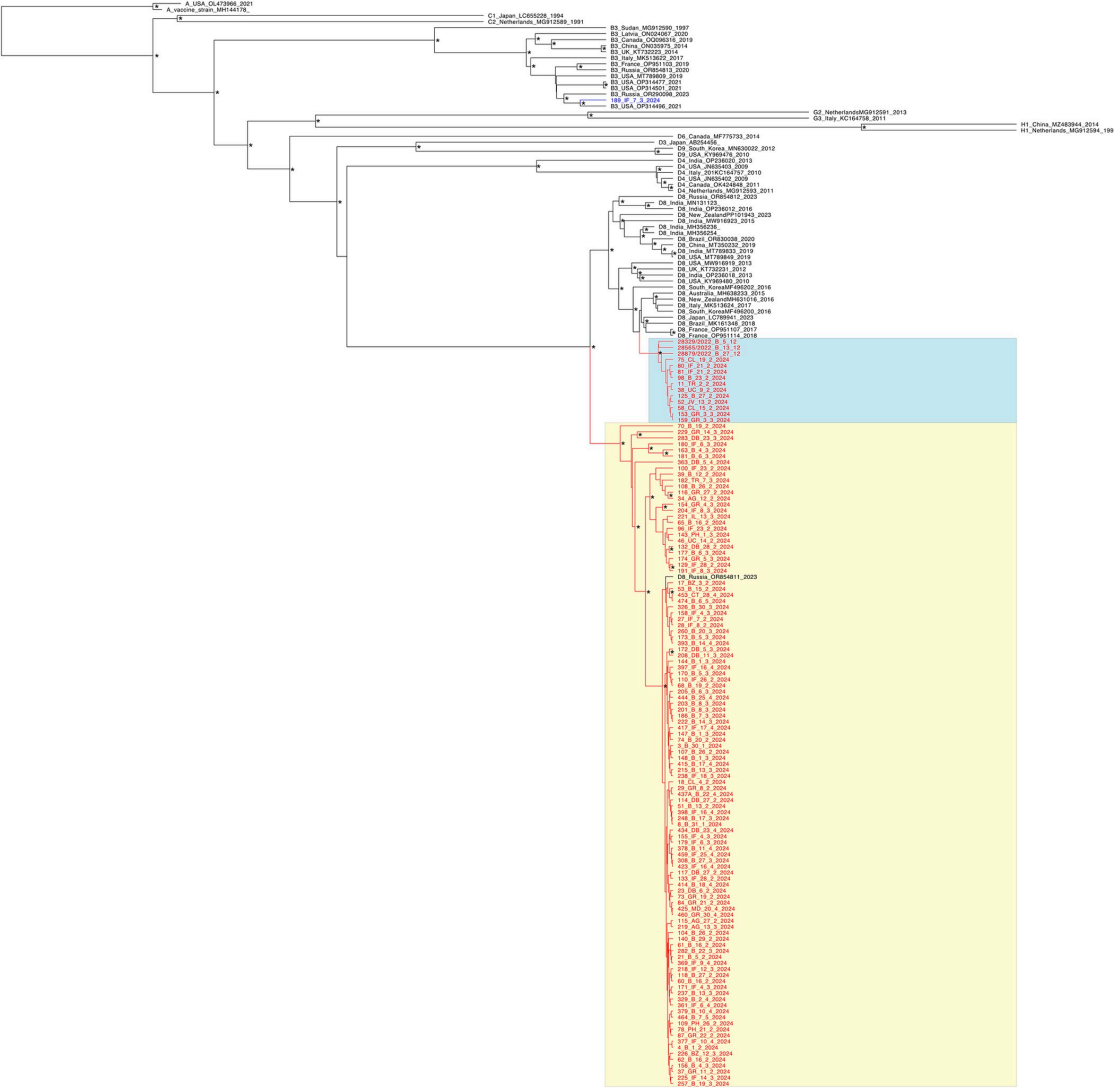
Phylogenetic tree of Romanian HMV whole genome sequences. The Romanian B3 genotype sequence is presented in blue, with the rest being shown with red branches. D8 genotype cluster 1 is highlighted in blue and cluster 2 in yellow. The clade specific reference strains are represented in black. * indicates node support values > 0.90.

WGS were generated for 124 out of 168 samples (73.8%). Seventy (56.5%) were from the paediatric population and 54 (43.5%) from adults. We have identified one B3 genotype sequence (189_IF_3_7_2024, SAMN43056117), whereas the rest were D8 genotype. The Romanian D8 HMV genomes are divided in two different well supported clusters: cluster 1, highlighted in blue, consisting of 14 sequences (11 cases from the spring of 2024, three from patients infected in late 2022) and cluster 2 (yellow) comprised 108 sequences (Fig 1).

Patient characteristics of the two clusters are summarised in Table 2. No significant differences were observed in epidemiological data, vaccination status, or the rate and type of associated complications between the clusters. However, the median duration of hospitalisation was 2 days longer for patients in cluster 1 compared to cluster 2 (p = 0.019).

**Table 2:** Patient characteristics according to cluster assignment.

Characteristics	Cluster 1, n (%)	Cluster 2, n (%)	p-value
	N = 14	N = 108	
**Epidemiological data**			
Children	10 (71.4)	59 (54.1)	0.219
Female	6 (42.9)	60 (55.0)	0.389
Urban	9 (64.3)	72 (66.1)	0.895
Epidemiological link	9 (64.3)	27 (24.8)	**0.002**
**Measles vaccination status**			
Unvaccinated	12 (85.7)	70 (64.2)	0.379
Incompletely vaccinated (1 dose)	0 (0.0)	7 (6.4)	
Fully vaccinated (2 doses)	0 (0.0)	7 (6.4)	
Unknown	2 (14.3)	25 (22.9)	
**Chronic conditions**			
At least one chronic disease	0 (0.0)	15 (13.8)	NA
**Complications**			
At least one complication	14 (100)	99 (90.8)	0.237
Conjunctivitis	12 (85.7)	82 (75.2)	0.384
Keratitis	1 (7.1)	26 (23.9)	0.155
Measles acute pneumonia	7 (50.0)	36 (33.0)	0.210
Acute respiratory failure	3 (21.4)	12 (11.0)	0.262
Acute hepatitis	0 (0.0)	28 (25.7)	NA
Myositis	2 (22.2)	33 (49.3)	0.127
Acute dehydration	8 (57.1)	43 (39.4)	0.206
Pericarditis	2 (14.3)	0 (0.0)	NA
Laryngitis	1 (7.1)	5 (4.6)	0.676
Pancreatitis	1 (7.1)	0 (0.0)	NA
Acute otitis acute	0 (0.0)	2 (1.8)	NA
Acute sinusitis	0 (0.0)	1 (0.9)	NA
Days of hospitalization, median (IQR)	7 (5, 7)	5 (4, 6)	**0.019**

NA–not applicable; IQR–interquartile range (25th, 75th percentile).

At the root of cluster 1 there is a cluster of sequences reported worldwide (2013–2017) with one reported in Italy in 2017, in Veneto region [13]. Cluster 2 is a large one, comprising sequences from all counties. Only one sequence from outside Romania is present within cluster 2 (Russia, OR854811).

In order to evaluate how informative different genomic regions of HMV are from a phylogenetic point of view, we have performed analysis on the HA gene, the partial nucleocapsid region (450nt) and the fusion gene. The phylogenetic trees are illustrated in S1–S3 Figs. Comparing the results of these analysis we concluded that the most informative genomic region for phylogeny was the HA, followed by the partial nucleocapsid (N). Both trees built on HA and N showed the same segregation of the Romanian HMV sequences in two clusters, but the analysis was less informative in terms of detecting local transmission networks compared to the full genome analysis. The weakest phylogenetic signal was observed when analysing the fusion gene. In this analysis, the topology of D8 clusters was lost.

### Variability in HA gene—differences between the two HMV Romanian clusters

**Fig 2:**
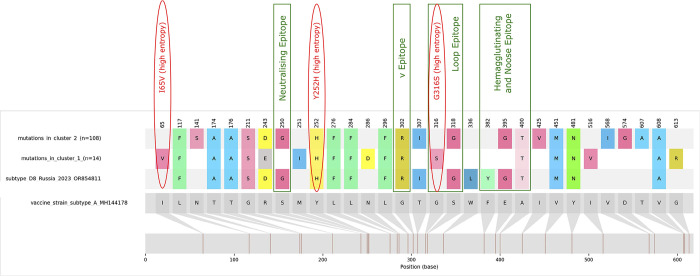
Amino acid differences within the HA gene between the two identified Romanian HMV clusters. In green: Positions within relevant epitopes [30]. In red: Mutations identified as having high entropy (described in Methods).

Comparing the Romanian sequence with the sequence from MMR vaccine (MH144178) and then D8 reference sequence (OR854811), we have identified several mutations that affected also the main HMV epitopes. We observed different mutation profiles specific to the two Romanian HMV clusters (Fig 2).

The mutation profile corresponding for HA gene of cluster 2 is more similar with D8 reference strain, presenting the several identical mutations from the vaccine strain. On the other hand, cluster 1 presented a distinct profile from cluster 2 and from the D8 reference strain used. Shannon Entropy showed statistical differences in particular positions (e.g. I65V, Y252H, G316S) (S4 Fig). The potential impact of the amino acids substitution found statistically different in the small transmission network is as follows:

i) I65V –this mutation would decrease the hydrophobicity in that region, since Valine is less hydrophobic compared to Isoleucine.

ii) Y252H –this substitution is right near the neutralizing epitope. Since Tyrosine is involved in stacking or pi-interaction with P263 and in H-bond with N262 from the neighbouring monomer, the histidine substitution may affect these interactions.

iii) G316S –this mutation might strengthen the local conformation by the presence of its sidechain, and therefore could increase the probability of H-bond interaction between Serine with other residues, whereas Glycine would increase flexibility in the region.

### Impact of N mutations on molecular surveillance

Recent reports suggested an accumulation of mutations in the N region, especially in D8 genotype, with possible impact on the sensitivity of the RT-qPCR test [31]. By analysing the 75nt targeted by CDC RT-PCR assay, we identify 4 distinct mutations. Two of them (C26T and T44G) are in the binding region of the probe and are found only in two distinct samples. The G71A substitution was exclusively found in cluster 1, whereas T65C is present in cluster 2 (S5 Fig).

## Discussions

This study provides an integrated analysis of the clinical, epidemiological, and virological characteristics of measles cases reported in southern Romania during 2024 epidemic. We analysed 168 nasopharyngeal samples from unique patients, monitored during hospitalization for measles symptoms and complications. WGS was successfully conducted on 124 samples. Although measles is typically a childhood disease [32,33], this outbreak also affected adults, with 37.5% of cases in our study being young adults (mean age 37.3 ± 8.7 years, maximum 56 years), many with chronic conditions (27.0%). By mid-July 2024, 2918 adult cases were reported in Romania, comprising 13.8% of the outbreak [8]. The ongoing measles outbreak in Romania has notably impacted adults, particularly young individuals with chronic conditions. This increased incidence of measles in aging cohort was reported also during 2016 epidemics and it was suggested to be the result of a longer transition to the vaccination program, waning of immunity in vaccinated people [34,35]. A previous report evaluating measles vaccine-induced immunity in England reported a slow rate waning, but sufficient to contribute to measles epidemic [35]. Only few of the adults included in this study declared that have been vaccinated with 2 doses while the majority were unvaccinated or did not know about their vaccination status. Romania has introduced MMR vaccination program in 2004 and reporting good coverage until 2010 [7,14,36,37].

Recognizing clinical characteristics early is crucial for implementing epidemiological measures to control this highly contagious infection [38]. In addition to classic measles symptoms like rash, fever, and cough, our study identified age-specific clinical features: children more commonly exhibited runny nose, red eyes, and diarrhoea, while adults more frequently experienced sore throat, headache, and myalgia. These findings, which differ from those in paediatric populations, provide an addition to the limited literature on adult measles manifestations [33,39,40]. Age-related differences in outcomes were also noted, with children more prone to ocular involvement, measles pneumonia, and dehydration, and adults more likely to suffer from liver involvement, as previously described [33].

Measles virus is one example of genetic variation within a monotypic virus: 24 HMV genotypes were identified so far and only one serotype [41]. During the last decade in Europe, the measles outbreaks have been mainly caused by two HMV genotypes, B3 and D8. B3 was mainly responsible for the previous extended measles outbreak in Romania, whereas D8 is now observed as driving this ongoing epidemic. Using HMV full genome sequences, the phylogenetic analysis offered interesting insights in the spreading of this virus in Romania. Genotype D8 is currently circulating both in adults and children. We have identified at least two imports events followed by local spread by the existence of two well supported transmission networks. The large cluster corresponding both for adults and children is now mainly responsible for the viral transmission in Romania. Only one external sequence was identified in this local large transmission cluster, one that has been reported in Moscow, Russia in 2023 (OR854811), suggesting a possible epidemiological link. The smaller D8 Romanian cluster consists of only 11 sequences from 2024. The HA analysis indicated distinct mutation profiles between these two clusters. In the small transmission network we have identified three sequences corresponding to viral strains that circulated in 2022 in Romania suggesting that this might be an older viral variant that is still circulating in Romania, but to a lesser extent. At the root of the smaller cluster, we have identified a cluster of sequences, one being reported in Italy in 2017, suggesting similarities with HMV strains from Veneto, possible related to the Romanian communities that are well represented in this region. However, cell culture experiments for evaluating the in vitro infectivity of these strains belonging to different phylogenetic clades are needed to investigate this hypothesis. Aside from the length of hospitalization, no significant differences were observed in the analysis of cases associated with the two clusters circulating in Romania.

The virus evolves through mutations that are naturally occurring and are selected through bottlenecks or under immune pressure. The analysis of HA sequences revealed the overall conserved nature of this gene, especially in the large Romanian transmission network that presented D8 specific mutations, whereas the small cluster had a different mutation profile. Both D8 variants identified in the present outbreak are harbouring mutations in N gene that may have a negative impact on the sensitivity of RT-PCR assay recommended by WHO for Measles molecular surveillance. D8 strains carrying mutations in the targeted region of this test were first reported in 2023 in Switzerland [31]. Therefore, it is important to perform sustained molecular surveillance of the measles strains to verify the current molecular diagnostic tests.

Romania is facing a troubling rise in vaccination scepticism, leading to a significant decline in vaccination rates among children and challenging the National Immunisation Program [42,43]. Previous studies reported very low vaccination rates in infants (around 50%) and even lower at the beginning of COVID-19 pandemic when travel restrictions were instituted [5]. Among the contributor factors to the low vaccination rate, Stanescu at al. identified beside vaccine hesitancy, the failure to attend general practitioner office, especially for the rural communities, missed doses due to temporary medical contraindications and loss in follow-up [7]. This situation highlights the urgent need to understand the clinical and molecular epidemiology in Romania and calls for robust public health interventions at both national and international levels to improve immunisation rates and control the ongoing measles outbreak.

There are several limitations of the study we have conducted. The time frame of this study is short and we have included patients with measles diagnostic that were treated in only one national centre during the spring of 2024, being representative for Southern Romania. However, the analysed samples were collected from the peak of the epidemic.

## Conclusions

Molecular epidemiology analysis indicated the co-circulation of two HMV variants in Romania in 2024. The epidemic is currently driven by the D8 genotype in particular; one large monophyletic cluster was identified suggesting local spread after one single/limited number of import events. The other Romanian HMV cluster is smaller and corresponds to patients that required a slightly longer hospitalisation period. Sustained genetic surveillance of this pathogen is recommended, as well as immune waning evaluation of vaccinated adults in order to improve vaccination programs in Romania, one of the most affected countries in this recent European measles outbreak. The ability to accurately track viral lineages via genetic sequencing is yet another useful tool to move towards the World Health Organization’s (WHO) goal of eliminating measles. Developing an adequate monitoring and surveillance capacity can lead to a better identification of transmission chains and therefore aid in the interruption of measles outbreaks.

## Supporting information

S1 Fig Phylogenetic analysis performed on hemagglutinin (HA) dataset. The blue branch represented the B3 genotype sequence, the Romanian D8 genotype sequences are represented in red. Cluster 1 is highlighted in blue and cluster 2 in yellow. The clade specific reference strains are represented in black.
(PDF)

S2 Fig Phylogenetic analysis performed on nucleocapsid (N) dataset. The blue branch represented the B3 genotype sequence, the Romanian D8 genotype sequences are represented in red. Cluster 1 is highlighted in blue and cluster 2 in yellow. The clade specific reference strains are represented in black.
(PDF)

S3 Fig Phylogenetic analysis performed on Fusion (F) dataset. The blue branch represented the B3 genotype sequence, the Romanian D8 genotype sequences are represented in red. Cluster 1 is highlighted in blue and cluster 2 in yellow. The clade specific reference strains are represented in black.
(PDF)

S4 Fig Cartoon representations of the superimposed X-ray crystallography structures of Measles virus hemagglutinin (HA) in complex with the SLAM receptor: A) top & side view of the monomeric 6-blade beta propeller structure of HA (PDB codes: 2rkc, 2zb5, 2zb6, 3alw) with epitopes highlighted; B) homo-tetrameric conformation of the HA (PDB code: 3alx) with mutated aminoacids’ sidechains shown as sticks.
(PDF)

S5 Fig Mutations identified N gene (75 nt) targeted by CDC RT-qPCR assay. Mutation positions are indicated on top.
(PDF)
