# Supplementary figures and images for: Ongoing measles outbreak in Romania: Clinical investigation and molecular epidemiology performed on whole genome sequences
Source: PLoS One. 2025 Jan 15;20(1):e0317045. doi: 10.1371/journal.pone.0317045 (PMC11734985; doi:10.1371/journal.pone.0317045)

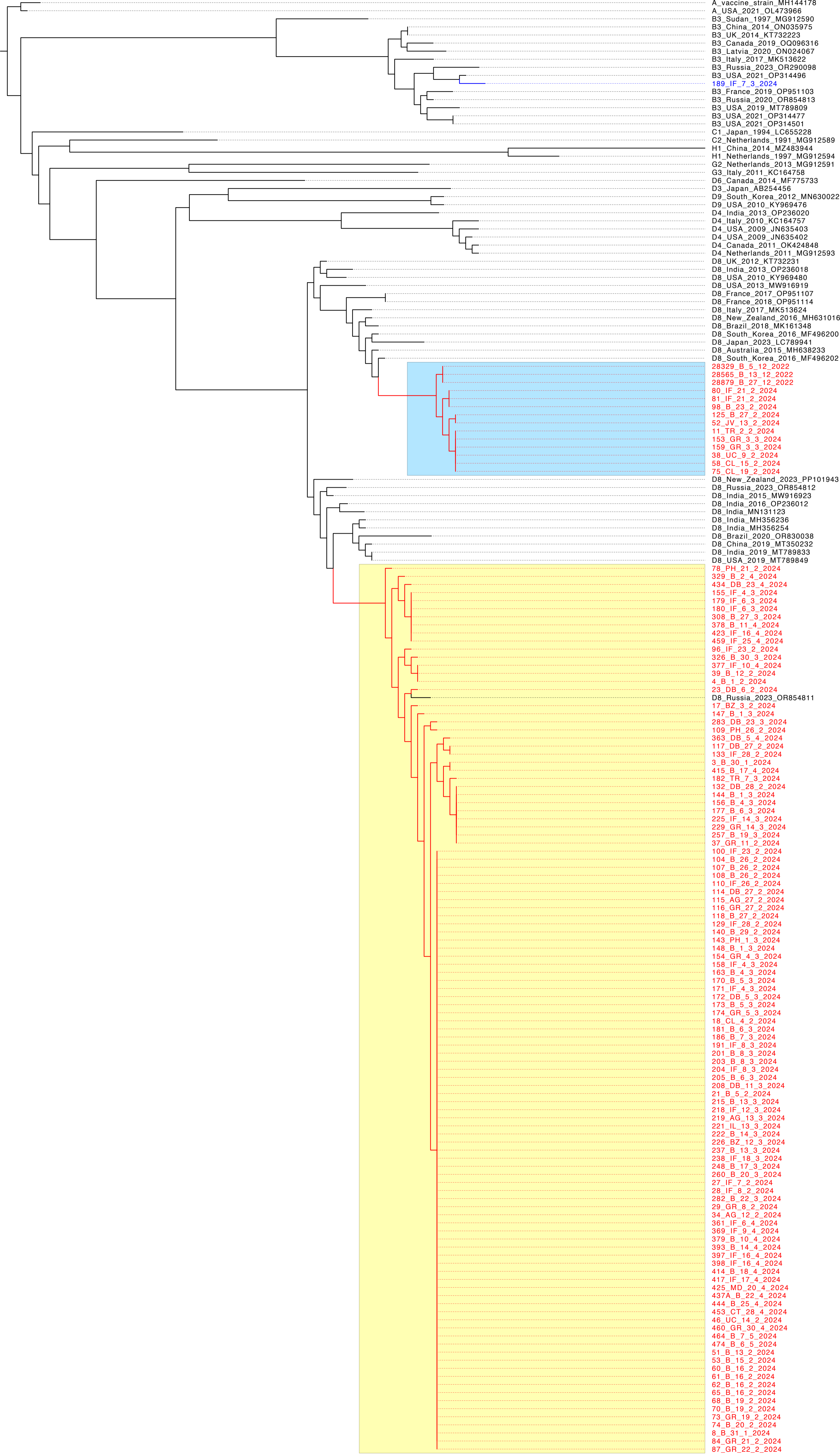

Supplement: S1 Fig — The blue branch represented the B3 genotype sequence, the Romanian D8 genotype sequences are represented in red. Cluster 1 is highlighted in blue and cluster 2 in yellow. The clade specific reference strains are represented in black. (PDF) [file pone.0317045.s001.pdf]

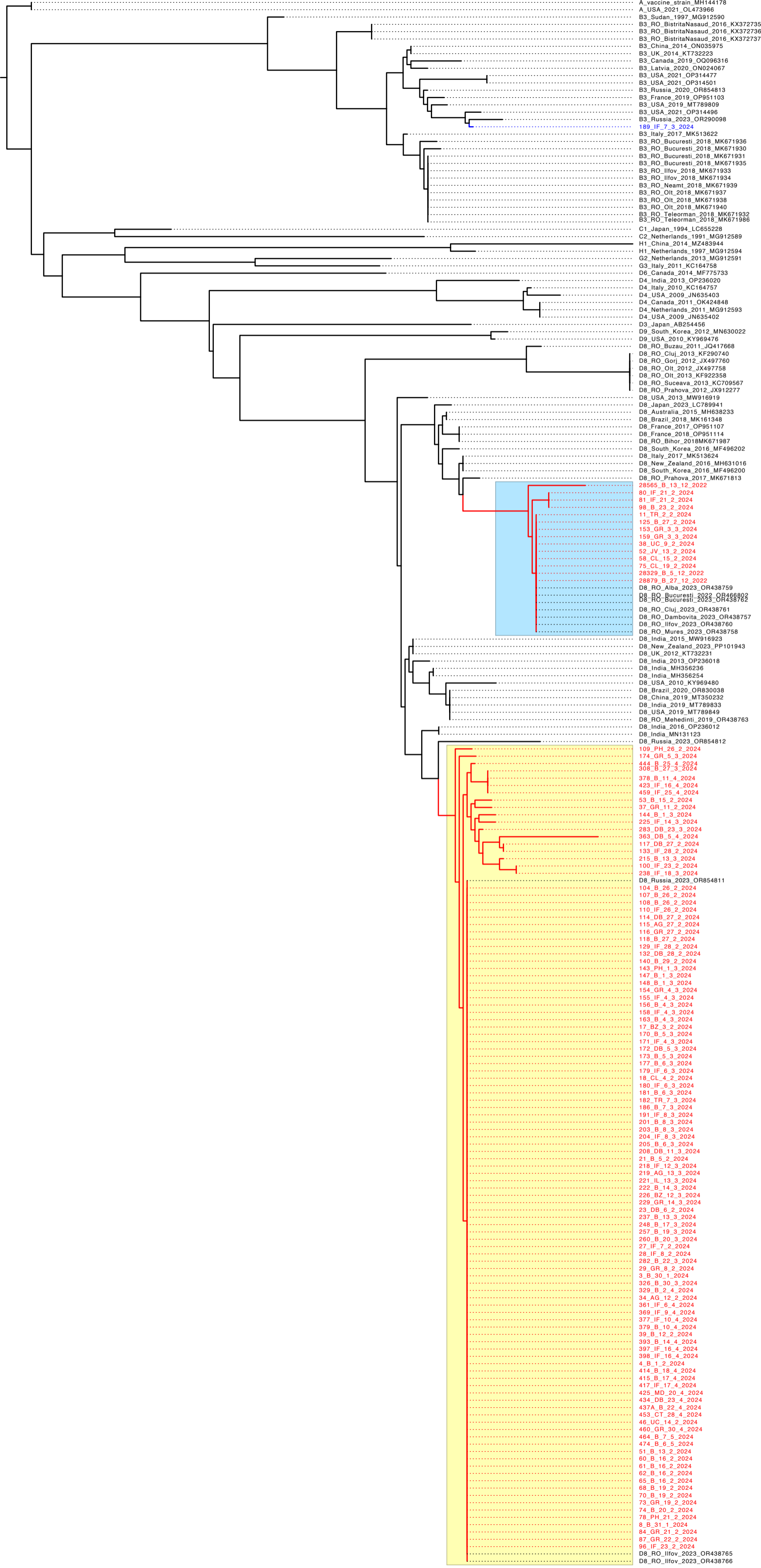

Supplement: S2 Fig — The blue branch represented the B3 genotype sequence, the Romanian D8 genotype sequences are represented in red. Cluster 1 is highlighted in blue and cluster 2 in yellow. The clade specific reference strains are represented in black. (PDF) [file pone.0317045.s002.pdf]

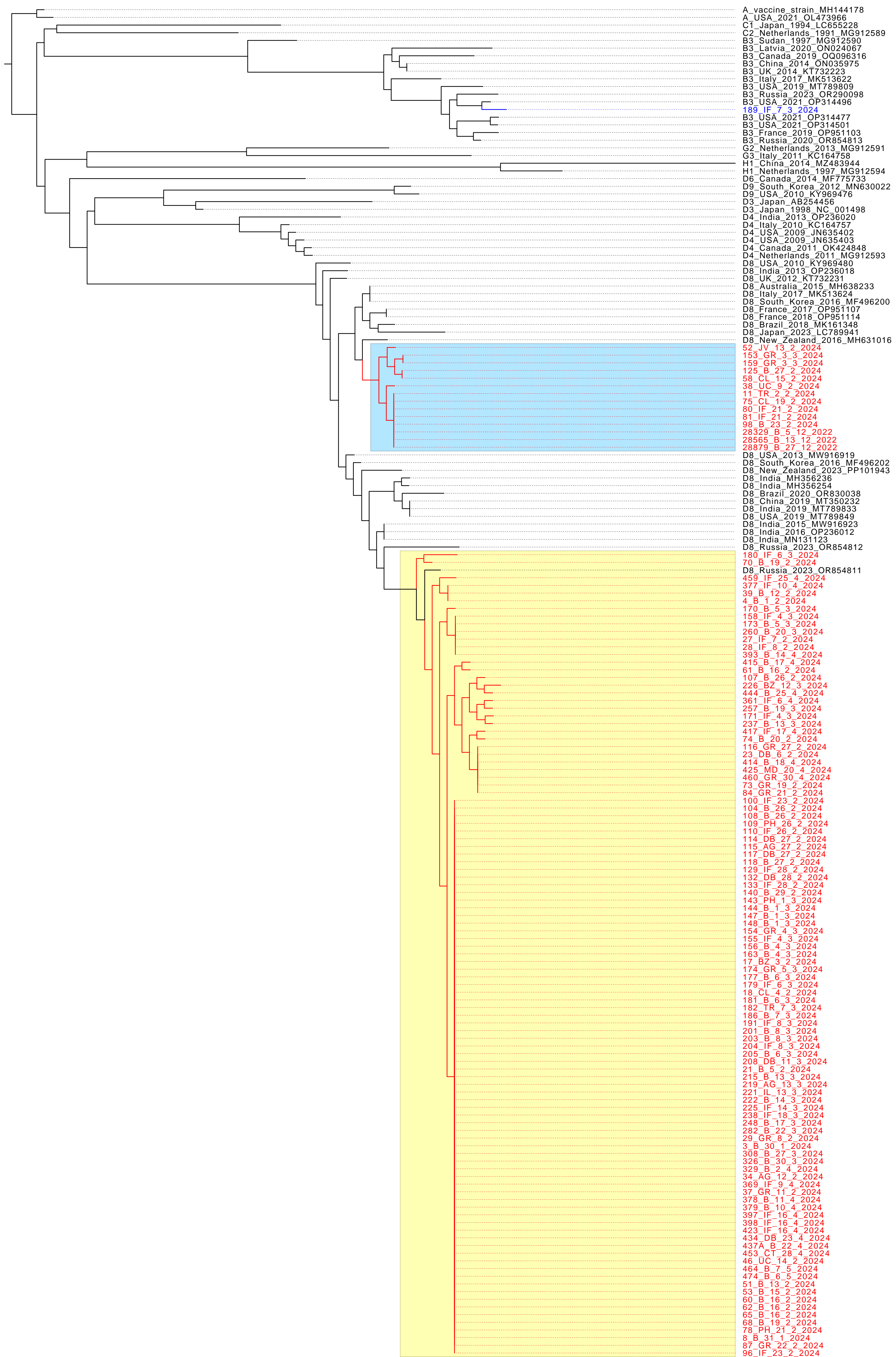

Supplement: S3 Fig — The blue branch represented the B3 genotype sequence, the Romanian D8 genotype sequences are represented in red. Cluster 1 is highlighted in blue and cluster 2 in yellow. The clade specific reference strains are represented in black. (PDF) [file pone.0317045.s003.pdf]

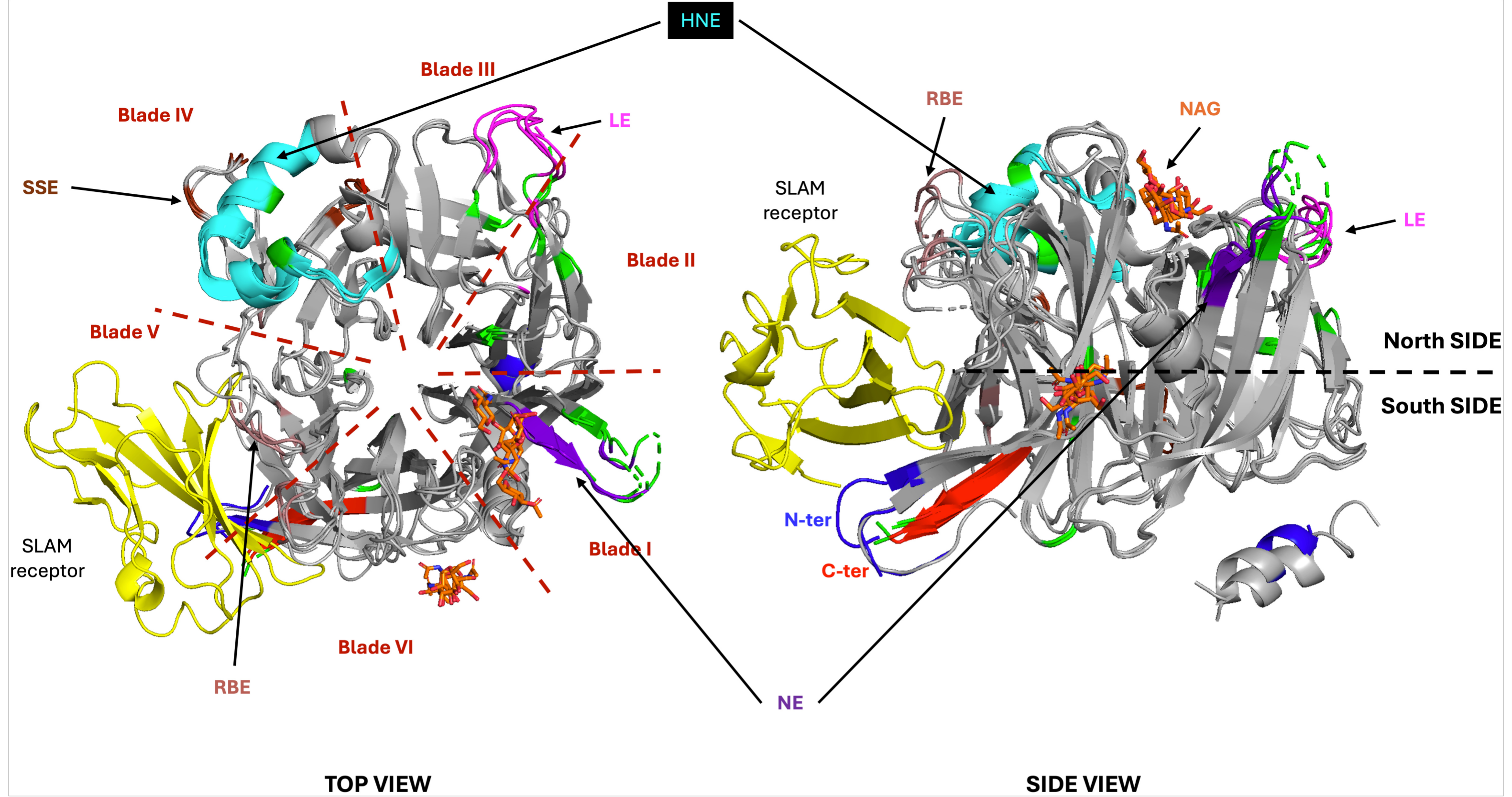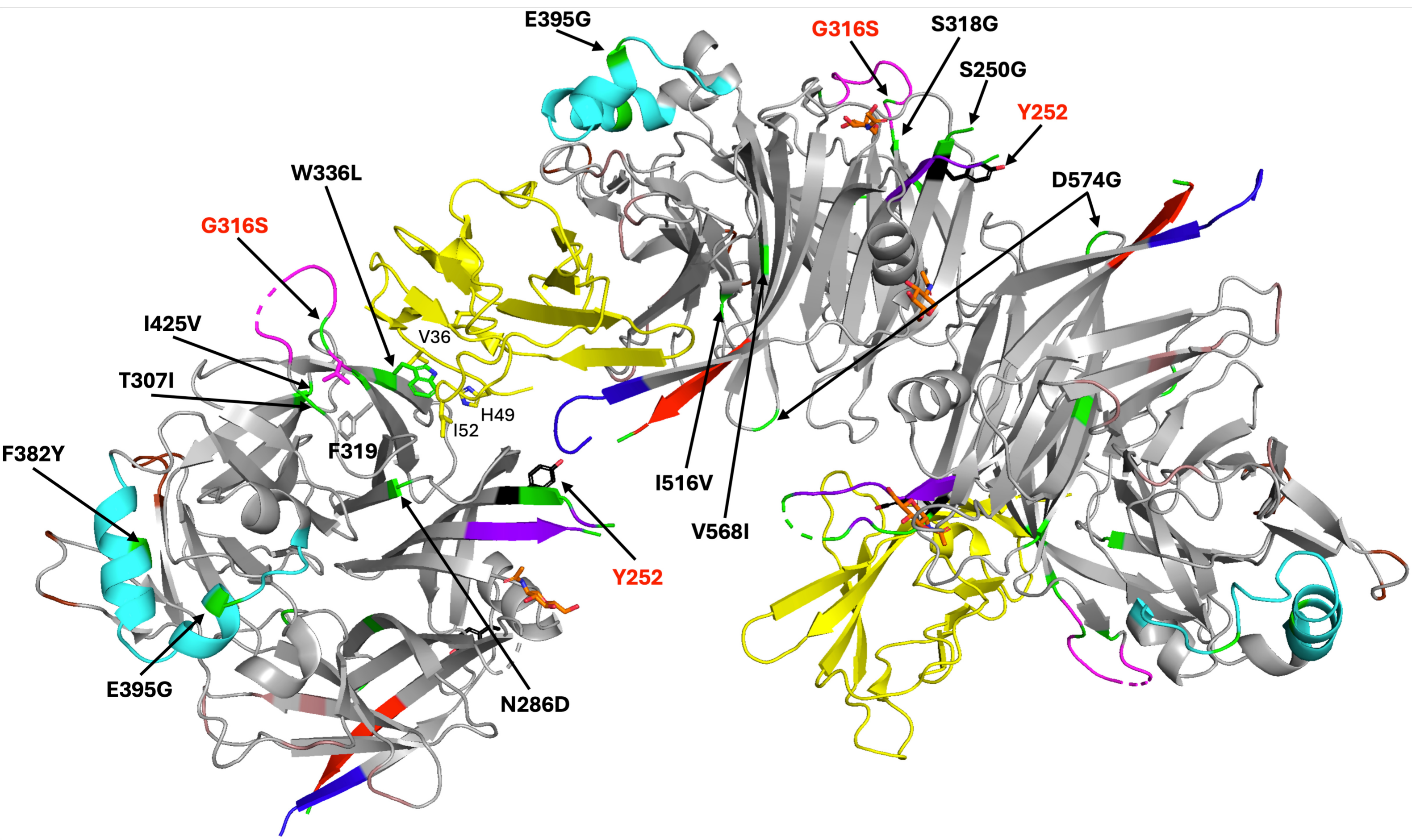

Supplement: S4 Fig — Cartoon representations of the superimposed X-ray crystallography structures of Measles virus hemagglutinin (HA) in complex with the SLAM receptor: A) top & side view of the monomeric 6-blade beta propeller structure of HA (PDB codes: 2rkc, 2zb5, 2zb6, 3alw) with epitopes highlighted; B) homo-tetrameric conformation of the HA (PDB code: 3alx) with mutated aminoacids’ sidechains shown as sticks. (PDF) [file pone.0317045.s004.pdf]

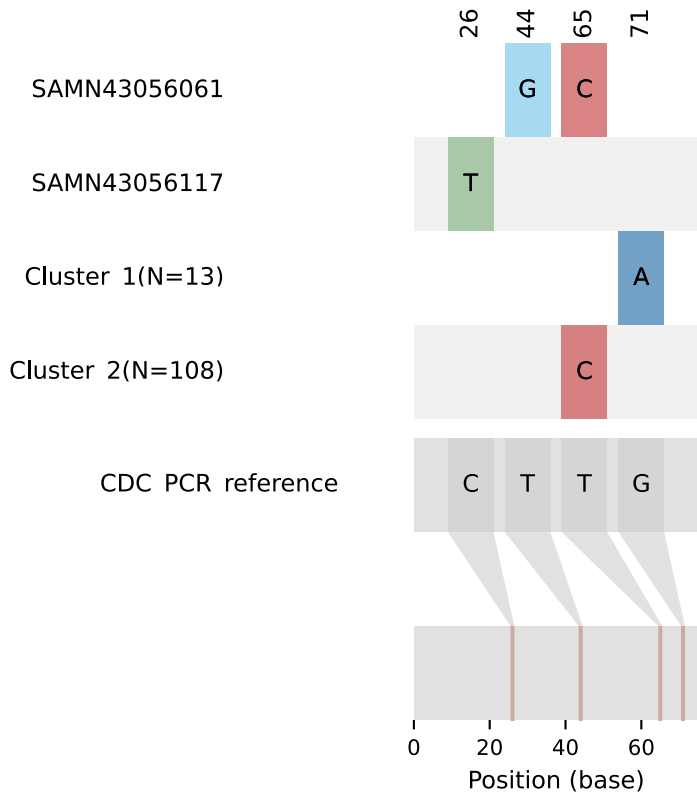

Supplement: S5 Fig — Mutation positions are indicated on top. (PDF) [file pone.0317045.s005.pdf]
